# Fatal acquired coagulation factor V deficiency after hepatectomy for advanced hepatocellular carcinoma as a possible immune checkpoint inhibitor-related adverse event: a case report

**DOI:** 10.1186/s40792-023-01601-2

**Published:** 2023-02-02

**Authors:** Shintaro Arakaki, Shinichiro Ono, Futoshi Kawamata, Shinichiro Ishino, Yasunori Uesato, Tomo Nakajima, Yukiko Nishi, Satoko Morishima, Shingo Arakaki, Tatsuji Maeshiro, Masayoshi Souri, Akitada Ichinose, Hiroaki Masuzaki, Mitsuhisa Takatsuki

**Affiliations:** 1 Department of Digestive and General Surgery, Graduate School of Medicine, University of the Ryukyus, 207 Uehara, Nishihara, Okinawa 903-0215 Japan; 2 Division of Endocrinology, Diabetes and Metabolism, Hematology, Rheumatology, Graduate School of Medicine, University of the Ryukyus, Nishihara, Japan; 3 Department of Infectious, Respiratory, and Digestive Medicine, Graduate School of Medicine, University of the Ryukyus, Nishihara, Japan; 4 The Japanese Collaborative Research Group (JCRG) on Autoimmune Coagulation Factor Deficiency (AiCFD), Japanese Ministry of Health, Labor, and Welfare (MHLW), Tokyo, Japan; 5 Department of Molecular Patho-Biochemistry and Pathobiology, Yamagata University School of Medicine, Yamagata, Japan

**Keywords:** Acquired coagulation factor deficiency, Hepatectomy, Immune checkpoint inhibitor-related adverse event

## Abstract

**Background:**

Atezolizumab plus bevacizumab therapy was recently introduced as the first line for unresectable advanced hepatocellular carcinoma (HCC), but immune-related adverse events (IrAEs) due to atezolizumab are a great concern. Here, we report the case of a patient who developed fatal acquired coagulation factor deficiency after hepatectomy for HCC, treated with atezolizumab and bevacizumab before surgery.

**Case presentation:**

A 70-year-old man received right trisegmentectomy of the liver with hepaticojejunostomy for advanced HCC with bile duct invasion, after atezolizumab and bevacizumab therapy. The patient suffered the sudden onset of severe multiple coagulation factor deficiency (II, V, VII, VIII, IX, X, XI and XII) immediately following reoperation for anastomotic leakage of hepaticojejunostomy, 7 days after hepatectomy. The coagulation factor deficiency did not reverse even with intensive treatment, and the patient died of uncontrollable bleeding 32 days after hepatectomy. An IrAE due to atezolizumab was suspected because the patient had developed the possible IrAE of enthesitis of the right gastrocnemius muscle before surgery, and specific inhibitors against factor V and anti-factor V autoantibodies were detected, leading to an ultimate diagnosis of autoimmune FV/5 deficiency (AiF5D).

**Conclusion:**

Severe acquired coagulopathy should be recognized as a possible life-threatening IrAE when using atezolizumab and bevacizumab for HCC.

## Introduction

Atezolizumab is an immune checkpoint inhibitor (ICI) against programmed death-ligand 1, and its combination with bevacizumab has recently been introduced as a novel and promising treatment for advanced hepatocellular carcinoma (HCC) [1, 2]. However, ICIs cause various immune-related adverse events (IrAEs), including acquired hemophilia [3–5], which is a great concern especially after hepatectomy. Here, we report a rare case of acquired factor V inhibitor (AFVI) that led to fatal uncontrollable bleeding, possibly due to an IrAE associated with atezolizumab and bevacizumab therapy for advanced HCC. The patient had the possible IrAE of enthesitis of the right gastrocnemius muscle before surgery, and developed uncontrollable coagulopathy after hepatectomy. The specific inhibitor against factor V was finally detected, leading to a diagnosis of AFVI, which may have been an IrAE due to atezolizumab in this case.

## Case presentation

**Fig. 1:**
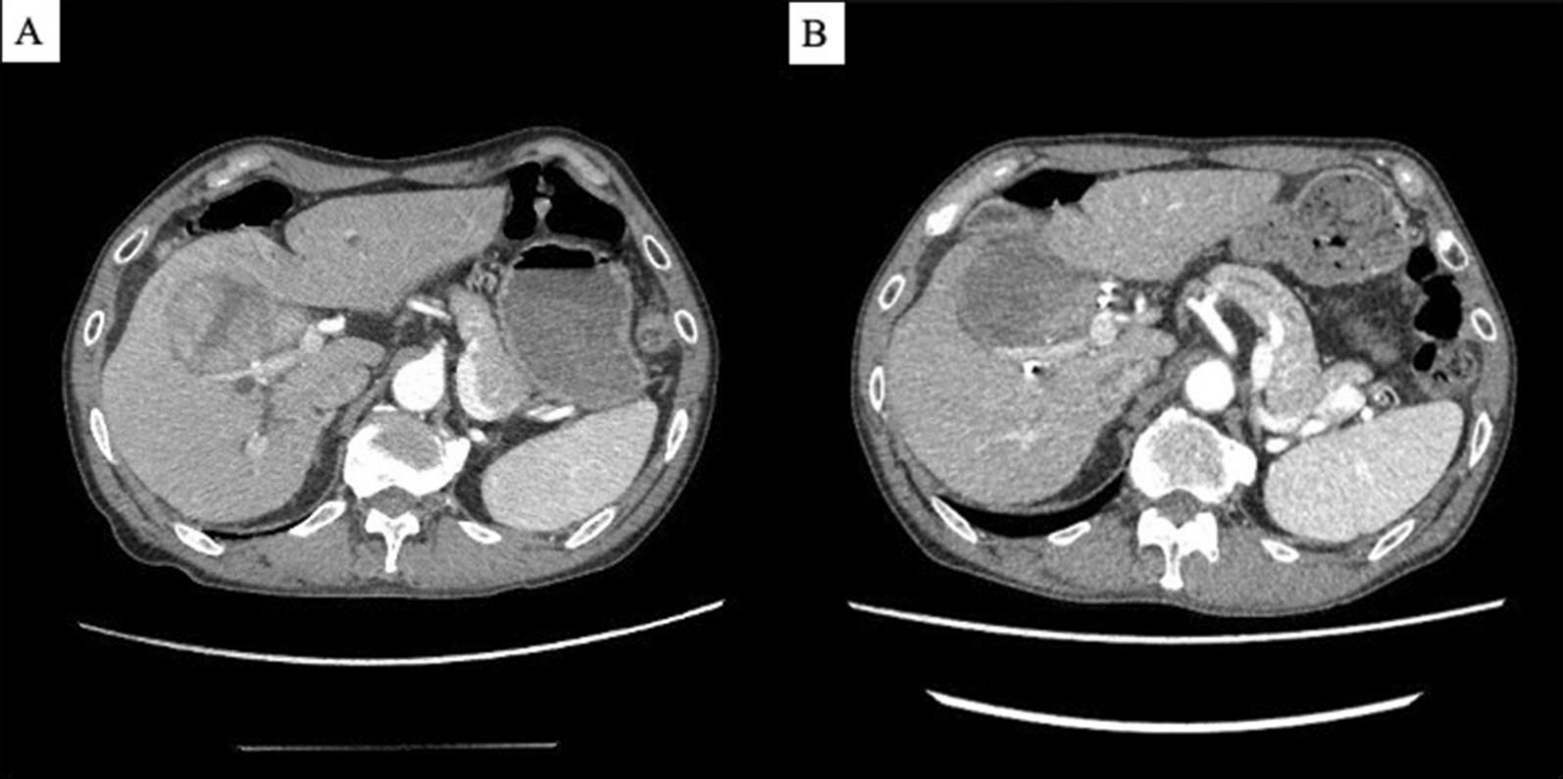
Preoperative contrast-enhanced computed tomography (CT) of the arterial phase. Huge hepatocellular carcinoma located in the hepatic hilum, before (**A**) and after (**B**) atezolizumab and bevacizumab treatment. The tumor viability seemed reduced; note that the CT attenuation number is diffusely decreased in **B**

A 70-year-old man was diagnosed with a solitary HCC 6 cm × 6 cm in size, located on the right side of the hepatic hilum, across the right anterior and medial segments (Fig. 1A). The patient suffered obstructive jaundice due to tumor invasion into the hilar bile duct, and first underwent endoscopic biliary stenting in the right hepatic duct. After his total bilirubin was normalized, we planned to perform right trisegmentectomy with hepaticojejunostomy of the liver. Although the tumor was estimated to be anatomically resectable, we introduced preoperative adjuvant chemotherapy with atezolizumab and bevacizumab to reduce tumor viability because the patient’s protein induced by vitamin K absence or antagonist II (PIVKA-II) level was remarkably high at 14,683 mAU/mL while his alpha-fetoprotein (AFP) level was normal (2.2 ng/mL). After six courses of the treatment, his PIVKA-II level had significantly decreased to 295 mAU/mL, and the viability of the tumor seemed to be decreased on a contrast-enhanced computed tomography scan that revealed defuse low-density change in the tumor (Fig. 1B). Although the liver function was good as Child–Pugh grade A with 0.934 of galactosyl human serum albumin (GSA) uptake ratio of the liver to the liver plus heart at 15 min (LHL) in technetium-99 m-GSA scintigraphy [6], portal vein embolization (PVE) of right lobe and segment 4 branches was performed because of the insufficient estimated remnant liver volume less than 30% (28.9%), and we finally could achieve the sufficient volume of 40.2%, 2 weeks after PVE. A preoperative blood test was normal except for mild anemia and a slightly low level of albumin, and the patient’s activated partial thromboplastin time (APTT) and prothrombin time (PT) were both normal (Table 1). During ICI treatment, the patient required prednisolone therapy for enthesitis of the right gastrocnemius muscle, which was suspected to be an IrAE due to atezolizumab. The symptom disappeared after the steroid treatment, and the patient underwent right trisegmentectomy of the liver with hepaticojejunostomy without blood transfusion, 8 weeks after the completion of atezolizumab and bevacizumab administration. Hepaticojejunostomy was performed between the confluence of segment 2/3 bile ducts and jejunum without biliary stent, because the bile duct was sick and enlarged due to the previous biliary obstruction, so that we believed biliary stent was not necessary.

**Table 1:** Preoperative data

Variable	Results	Reference range
WBC (/μL)	6.2 × 10^3^	3.3–8.6 × 10^3^
RBC (/μL)	4.55 × 10^6^	4.35–5.55 × 10^6^
Hb (g/dL)	12.7	13.7–16.8
PLT (/μL)	222 × 10^3^	158–348 × 10^3^
ALB (g/dL)	3.4	4.1–5.1
T-Bil (mg/dL)	1.1	0.4–1.5
AST (U/L)	21	13–30
ALT (U/L)	17	10–42
APTT (s)	28.2	23.5–35.0
PT (%)	93.4	70.0–130.0
PT INR	1.03	0.85–1.30
AFP (ng/mL)	< 2	0.0–14.9
PIVKA-II (mAU/mL)	295	0–39

*WBC* white blood cell, *RBC* red blood cell, *Hb* hemoglobin, *PLT* platelet, *ALB* albumin, *T-Bil* total bilirubin, *AST* aspartate aminotransferase, *ALT* alanine aminotransferase, *APTT* activated partial thromboplastin time, *PT* prothrombin time, *INR* international normalized ratio, *AFP* alfa-fetoprotein, *PIVKA-II* protein induced by vitamin K absence or antagonist-II

**Fig. 2:**
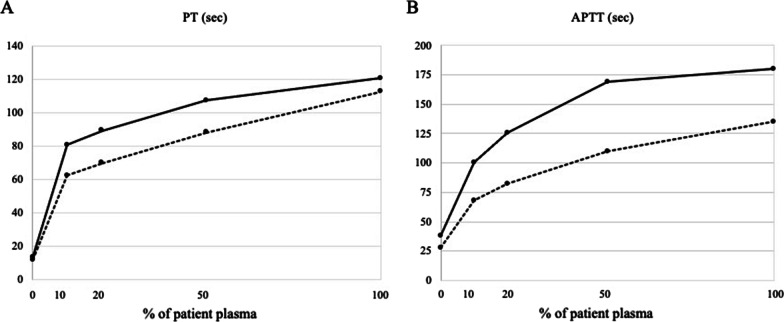
Results of a cross-mixing test using normal stocked plasma. The immediate reaction and that after 2 h show a convex graph as the inhibitor pattern both in prothrombin time (**A**) and in activated partial thromboplastin time (**B**)

The patient’s postoperative course was uneventful until he developed anastomotic leakage of both the hepaticojejunostomy and the jejunojejunostomy, which required urgent surgery 7 days after hepatectomy. Both were re-anastomosed, and a 2.5-mm plastic biliary stent was placed during the hepaticojejunostomy. At 9 days after hepatectomy (2 days after reoperation), although the patient appeared to have been doing well, laboratory data showed a sudden onset of significant prolongation of APTT (109.7 s) and the PT international normalized ratio (> 7.02). An initial assay of coagulation factors revealed that factors II, V, VII, VIII, IX, X, XI and XII were all significantly decreased (Table 2), though only factors V and VII were below normal range after the serum sample was diluted fourfold (Table 3). Lupus anticoagulant could not be calculated, and anti-cardiolipin-beta2-glycoprotein I complex antibody was not detected (Table 4). The previous anastomotic leakage was well controlled without recurrence, and no other surgery-related complications were observed. At the onset of coagulopathy, the level of total bilirubin was 1.5 mg/dL, creatinine was 1.86 mg/dL, C-reactive protein was 8.66 mg/dL, white blood cell count was 10,500/μL and platelet count was 68,000/μL. Acquired coagulation factor deficiency due to immunologic destruction was suspected because a cross-mixing test (CMT) revealed an inhibitor pattern (Fig. 2A, B). After the first surgery of hepatectomy, the antibiotics were administrated as follows; cefmetazole 1 g × 3 per day for 3 days, tazobactam sodium/piperacillin sodium 4.5 g × 3 per day for next 4 days, and meropenem 1 g × 2 per day, daptomycin 350 mg × 1 per day after reoperation, until the patient died. Meropenem and daptomycin were used for *Pseudomonas aeruginosa* and *Enterococcus faecium*, both of which were identified in the abdominal fluid in the drain the day after the second operation, and there was no severe peritonitis nor bacteremia throughout the course.

**Table 2:** Coagulation data at the onset of coagulopathy

Variable	Results	Reference range
Prothrombin time		
(s)	104.2	10.0–15.0
(%)	< 7.0	70.0–130.0
PT-INR	> 7.02	0.85–1.30
Activated partial thromboplastin time (s)	123.9	23.5–35.0
Fibrinogen (mg/dL)	465	180–350
D-dimer (μg/mL)	6.1	0.0–1.0
Fibrin/fibrinogen degradation products (μg/mL)	9	0–5
Factor II activity (%)	< 6	70–120
Factor V activity (%)	< 6	70–140
Factor VII activity (%)	< 6	70–120
Factor VIII activity (%)	64	70–130
Factor IX activity (%)	11	70–120
Factor X activity (%)	< 6	70–120
Factor XI activity (%)	< 1	70–120
Factor XII activity (%)	21	70–150
Factor XIII activity (%)	87	70–130
Lupus anticoagulant		
SCT (screening) (s)	≥ 300	
SCT (confirmatory) (s)	≥ 240	
The normalized SCT ratio	N.D	< 1.16
Anti-cardiolipin-beta2-glycoprotein I complex antibody (U/mL)	≤ 1.2	< 3.5

**Table 3:** Coagulation factor activity at the onset of coagulopathy (%)

	Dilution 1×	Dilution 4×
II	< 6	44.4
V	< 6	< 22.0
VII	< 6	< 22.8
VIII	64	166
IX	11	64
X	< 6	40
XI	< 1	13.7
XII	21	43.7
XII	87	–

**Table 4:** Results of JCRG’s Integrated Consignment Screening Test at postoperative day 16

Variable	Results	Reference range
a_2_-Plasmin inhibitor (%)	63%	85–115
PIC (mg/mL)	< 0.3 mg/mL	< 0.8
FDP (mg/mL)	19 mg/mL	< 4
D-dimer (mg/mL)	13.56 mg/mL	< 1.0
Fibrinogen (mg/dL)	158 mg/dL	150–400
FXIII/13 antigen (%)	59%	70–140
VWF antigen (%)	205%	50–155
Plasminogen (%)	51%	75–125
E-XDP (mg/mL)	38.6 mg/mL	–
Total PAI (ng/mL)	99 ng/mL	< 50
FXIII/13 activity (%)	69%	70–140
VWF Rco activity (%)	272%	60–170
FV/5 activity (%)	< 3%	70–135
FVIII/8 activity (%)	< 1%	60–150
FX/10 activity (%)	< 3%	70–130

*PIC* plasmin–plasmin inhibitor complex, *FDP* fibrin/fibrinogen degradation products, *VWF* von Willebrand factor, *E-XDP* elastase-digested cross-linked fibrin degradation products, *PAI* plasminogen activator inhibitor type-I, *Rco* ristocetin cofactor

**Fig. 3:**
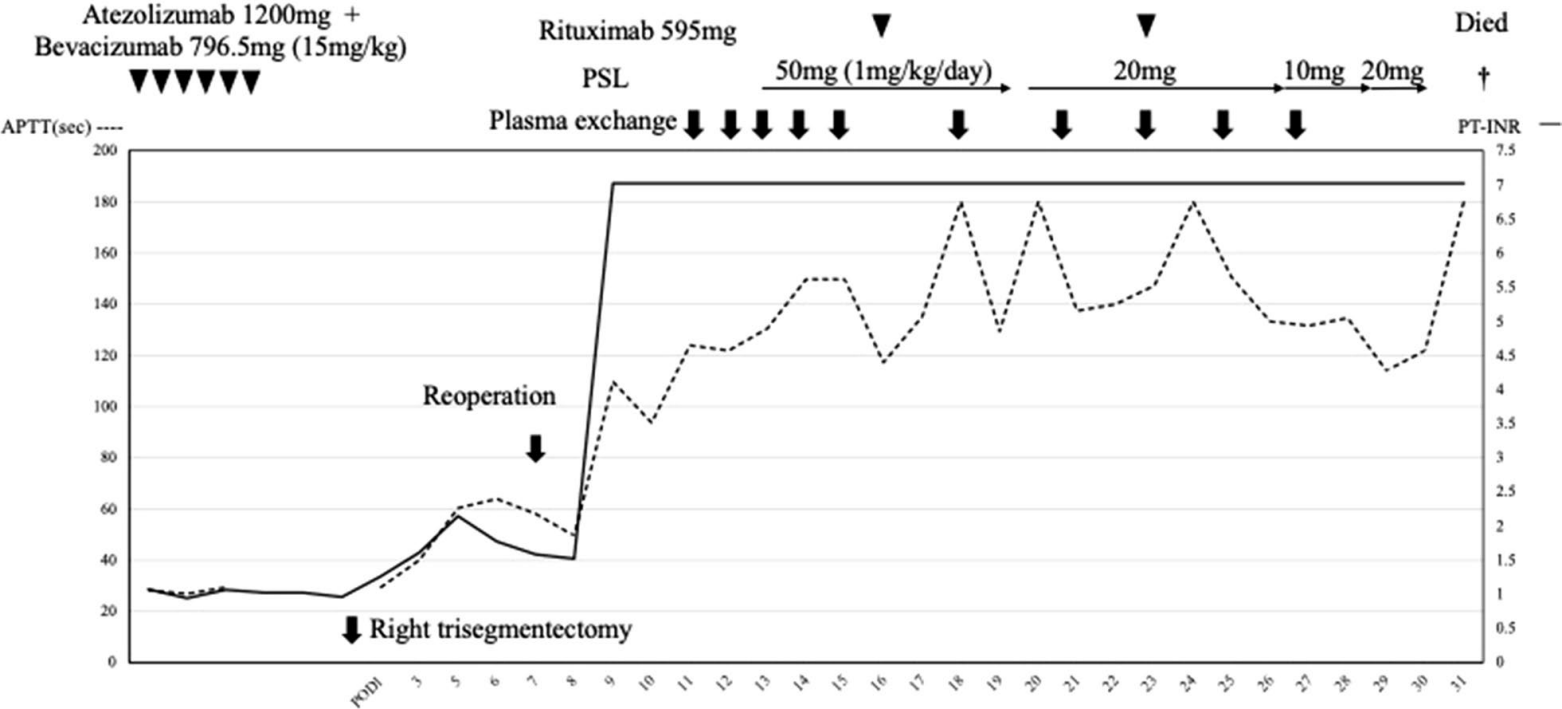
Perioperative course. The solid line indicates the change in prothrombin time, while the dotted line indicates change in the activated partial thromboplastin time. Before surgery, 6 courses of atezolizumab (1200 mg) plus bevacizumab (796.5 mg [15 mg/kg]) were administered with 21-day intervals between courses. Right trisegmentectomy of the liver was performed 8 days after completion of atezolizumab and bevacizumab administration. *POD* postoperative day

**Fig. 4:**
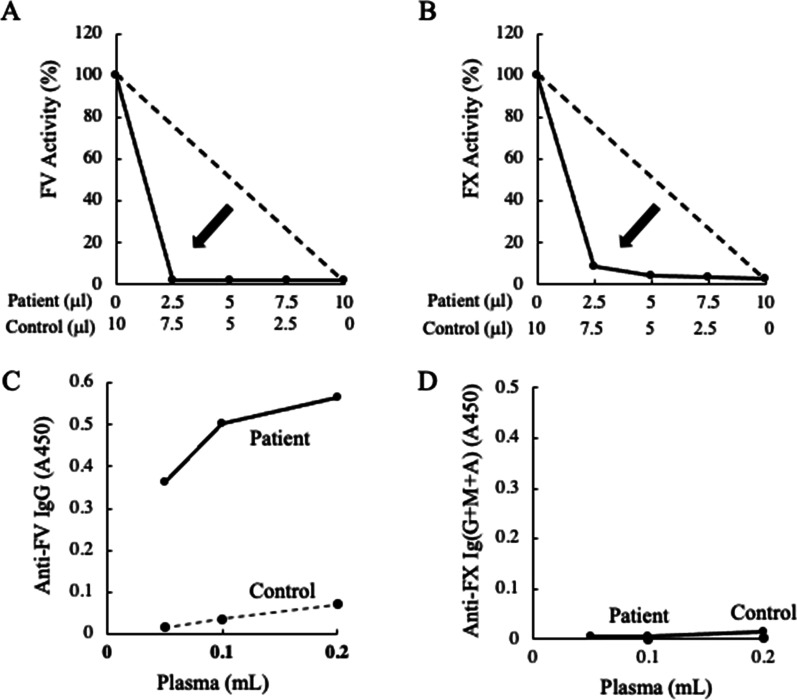
Results of the Japanese Collaborative Research Group’s detailed experimental examination after the patient died. FV/5-specific (**A**) and FX/10-specific (**B**) cross-mixing tests measured residual FV/5 and FX/10 activities, respectively. Anti-FV/5 autoantibodies (**C**) and anti-FX/10 autoantibodies (**D**) were measured by in-house enzyme-linked immunosorbent assay using purified human FV/5 and FX/10, respectively, as previously described [9]

Once we suspected immune-mediated coagulopathy, the patient was given intensive treatment in the form of repeated plasma exchange, 2 courses of rituximab and a steroid bolus. Even after rituximab administration, however, the coagulopathy was not reversed, and the patient died of uncontrollable systemic bleeding 25 days after the onset of coagulopathy (32 days after hepatectomy) (Fig. 3). After the patient died, a stored blood sample was sent to the Japanese Collaborative Research Group for an Integrated Consignment Screening Test [7, 8], and detailed experimental examination of stored patient plasma collected at postoperative day 16, confirmed severe multiple coagulation factor deficiencies, including deficiencies in Factor V/5 (FV/5), Factor X/10 (FX/10), and Factor VIII/8 (FVIII/8) (Table 1). Unfortunately, not all items (e.g., antithrombin, thrombin–antithrombin complex, etc.) were examined, due to the limited amount of plasma available. Both the FV/5-specific CMT and the FX/10-specific CMT showed a downward convex inhibition pattern (Fig. 4A, B), and the titers of FV/5 and FX/10 inhibitors were > 100 BU/mL and 2.5 BU/mL, respectively. However, an in-house enzyme-linked immunosorbent assay detected only anti-FV/5 autoantibodies, but not anti-FX/10 autoantibodies (Fig. 4C, D). Accordingly, the patient had true FV/5 inhibitor and false FX/10 inhibitor [8]. These findings are consistent with the definitive diagnosis of autoimmune FV/5 deficiency (AiF5D) according to the Japanese government criteria established by the Ministry of Health, Labor and Welfare [9]. Our ultimate diagnosis of this refractory coagulopathy was AiF5D possibly due to an atezolizumab-related IrAE.

## Discussion

In the present case, we first suspected disseminated intravascular coagulation (DIC) related to various pathologies as the cause of coagulopathy. However, this was excluded because the patient’s fibrinogen was rather elevated, his coagulopathy was too severe and had developed independently without liver/kidney failure, and there were no severe systemic infections. Although there was anastomotic leakage of the hepaticojejunostomy and jejunojejunostomy, it was successfully managed with reoperation. It is difficult to determine the definitive cause of severe coagulation factor deficiency in this case, however, we strongly suspect an IrAE, specifically the immune-mediated destruction of coagulation factors, because CMTs revealed inhibitor patterns in both PT and APTT. Inflammation caused by anastomotic leakage might have triggered this immune reaction as a secondary reaction following the possible IrAE of preoperative enthesitis of the right gastrocnemius muscle. Our proteome analysis indicated that inflammation may trigger the onset of autoimmune coagulation factor deficiencies, including AiF5D [10]. The patient had no other risk factors related to the development of such a severe immune reaction, nor any history of autoimmune disease.

Acquired coagulation factor inhibitor is recognized primarily as acquired hemophilia A, as a single inhibitor for coagulation factor VIII, and its possible causes include the use of ICIs [3–5]. AiF5D is extremely rare, with an estimated incidence of 0.038 per million persons per year [11]. Exposure to topical bovine thrombin has been identified as a possible cause [12], but it was not used in the present case. The use of antibiotics has also been cited as a cause [12], and regarding ICIs, Kida et al. presented the case of a patient who developed AiF5D after nivolumab administration for recurrent hypopharyngeal cancer, 4 months after tumor resection [13]. In that report, the patient showed no significant bleeding tendency, and overall, although the severity of bleeding varied, the majority of cases remitted with or without inhibitor persistence [12]. Actually, the first surgery of hepatectomy could be the trigger, but the clinical course was uneventful until the anastomotic leakage occurred, so that we believed that the patient was well controlled with antibiotics and drainage and inflammation was not severe enough to activate the immune reaction. Also, as mentioned above, antibiotics could be the trigger, and meropenem and/or daptomycin were the most possible candidate in the current case because the sudden onset of coagulopathy developed just after the reoperation. We definitively could not exclude the possibility of them, but we strongly suspect that IrAE was the most conceivable cause of severe and fatal immune-mediated coagulopathy in the current case, because there was no report of such severe and uncontrollable AiF5D due to other causes as mentioned above.

The therapeutic strategy for autoimmune acquired coagulopathy is the administration of corticosteroids in combination with immunosuppressive therapies (rituximab or cyclophosphamide), together with plasma exchange [12]. In our case, we performed plasma exchange 10 times and administered 2 courses of rituximab and a steroid bolus, but the coagulation factor deficiency did not reverse, possibly because the rituximab was administered too late, or it may be that autoantibodies (rather than autoreactive T cells or B cells) may contribute to the persistence of AiF5D.

In conclusion, although the combination of atezolizumab and bevacizumab is generally safe and is a promising treatment for advanced HCC, life-threatening IrAEs may occur. When we consider conversion surgery for advanced HCC, we must keep in mind the possibility of the unexpected and severe complication of AiF5D.

## Data Availability

All data generated or analyzed during this study are included in this article. Further inquiries can be directed to the corresponding author.

## Abbreviations

HCC: Hepatocellular carcinoma
IrAE: Immune-related adverse event
AiF5D: Autoimmune factor V deficiency
ICI: Immune checkpoint inhibitor
AFVI: Acquired factor V deficiency
PIVKA-II: Protein induced by vitamin K absence or antagonist II
AFP: Alfa-fetoprotein
APTT: Activated partial thromboplastin time
PT: Prothrombin time
CMT: Cross-mixing test
FV/5: Factor V/5
FX/10: Factor X/10
FVIII/8: Factor VIII/8
AiF5D: Autoimmune factor V/5 deficiency
DIC: Disseminated intravascular coagulation
